# Methodologic considerations on how to identify human hematopoietic stem cells

**DOI:** 10.1016/j.exphem.2025.104729

**Published:** 2025-04

**Authors:** Taylor Hinchly, Dominique Bonnet, Fernando Anjos-Afonso

**Affiliations:** a Haematopoietic Signalling Group, European Cancer Stem Cell Institute, School of Biosciences, Cardiff University, Cardiff, United Kingdom; b Haematopoietic Stem Cell Lab, The Francis Crick Institute, London, United Kingdom

## Abstract

Recently, human CD34^+^ hematopoietic stem cells (HSCs) have been purified to a frequency of approximately one in three cells, a population denoted as CD34^+^CD38^−^CD45RA^−^CD90^+/−^ endothelial protein C receptor (EPCR)^+^ HSCs. This work aimed to evaluate the methodology for CD34^+^ HSC isolation, exploring differences in antibody clones, conjugates, source of cells, and additional cell surface antigens (integrin-α6, CLEC9A, and GPRC5C) to enhance the purity of these EPCR^+^ HSCs. We are emphasizing here the importance of experimental planning and antibody panel selection concerning the isolation of these human HSCs from multiple sources and providing important notes on the pitfalls of the reagents used for such purposes. Our results should enable a better reproducibility of results between laboratory tests as well as further pursuits of work toward improving the enrichment of human HSCs.

## Highlights

• Flow-cytometry methods for human CD34^+^ HSC identification are reported.
• Antibody selection includes clones and fluorochromes.
• Integrin-a6 (CD49f), EPCR (CD201), CLEA9A (CD370), and GPRC5C are described.

The hematopoietic system is highly regenerative and is organized hierarchically with hematopoietic stem cells (HSCs) sitting at the apex. Despite being some of the most widely studied stem cells, questions remain regarding their activity and regulation, in particular in the human setting. Such work is limited by our ability to isolate these rare human cells as they reside within a highly heterogeneous pool of stem and progenitor cells. Following the identification of the sialomucin protein CD34 as a marker of hematopoietic stem and progenitor cells (HSPCs) [1], further work aimed to better enrich these cells. This included the use of other cell surface antigens, such as CD38 [2], CD90 [3], CD45RA [4], and CD49f [5]. Combining all of these markers, it was possible to refine the population with a stem cell frequency of approximately 1 in 10 cells [5].

Recently, work within our laboratory purified CD34^+^ HSCs to a frequency of approximately one in three cells, with the addition of endothelial protein C receptor (EPCR) expression as a selection marker, described as CD34^+^CD38^−^CD45RA^−^CD90^+/−^EPCR^+^, hereafter EPCR^+^ HSCs [6], the most well-purified human HSC population to date. Other groups have also trialed alternative cell surface antigens to enhance the isolation of human HSCs including CLEC9A (c-type lectin domain containing 9A or CD370) and GPRC5C (G protein-coupled receptor class C group 5 member c) among others [7,8]. Our initial aim was to combine all these recently described antigens to further refine the EPCR^+^ HSC population; however, during this process, we encountered some limitations in using them. Therefore, we decided to report here some methodologic notes on how to exploit CD49f, CD201 (EPCR), CD370 (CLEC9A), and GPRC5C as cell surface antigens to describe human HSCs, including appropriate clones and conjugates of the antibodies and different sources of HSPCs, such as cord blood (CB) or bone marrow (BM). This work aimed to provide important notes for those in the field to better isolate and by extension assess human HSCs and to notify some of the pitfalls of the reagents used for such purposes.

## METHODS

### Cell Processing

Briefly, as previously reported [6], CB was obtained after informed consent from the Royal London Hospital (REC: 06/Q0604/110) and the University Hospital of Wales (REC: 06/WSE03/6). The protocols were approved by their respective ethical committee. Mononuclear cells (MNCs) were also purchased from StemCell Technologies. Human BM-MNCs were purchased from Lonza Biologics and StemCell Technologies. Informed consent was obtained in accordance with the Declaration of Helsinki. BM1 (25 million MNCs) was from a woman, 54-year-old African-American donor, and BM2 (25 million MNCs) was from a man, 36-year-old Caucasian donor. For most experiments, 3–5 CB samples were pooled for each experiment (100–200 million MNCs) and enriched CD34^+^ (eCD34^+^) cells were positively selected using the EasySep CD34^+^ selection kit (StemCell Technologies) according to the manufacturer's instructions with only 5 rounds of immunomagnetic separation. Typically, after magnetic separation 1–2 × 10^6^ eCD34^+^ were obtained, and cells were divided into separate immunophenotype stains, each containing 1.25–2.5 × 10^5^ eCD34^+^ cells. Individual CB donors (*n* = 8; 20–40 million MNCs) were also used for certain experiments. After magnetic separation 1.25–2.5 × 10^5^ eCD34^+^ were obtained.

### Immunostaining

In general, a maximum of 1 × 10^6^ cells were incubated with antibodies (see Table 1) for 30 min at 4°C in 50 μL of staining buffer (phosphate buffer saline [PBS] with 2% of fetal bovine serum [FBS]) containing HGG (human γ-globulins from Cohn fraction II, III; Sigma-Merck) at 1:5 final dilution (from a 20 mg/mL stock) as blocking reagent. When biotin-conjugated primary antibodies were used, the appropriate streptavidin binding step was used with washes performed between incubations. After the incubations the cells were washed with staining buffer and resuspended in staining buffer containing 4′,6-diamidino-2-phenylindole (DAPI; 1:2,000 from a 200 μg/mL stock; Merk-Sigma) before analysis on an LSRFortessa (BD Biosciences). In case fewer than 0.5 × 10^6^ cells were used, the staining was performed in 25 μL of staining buffer containing half of the amount of the antibodies as suggested in Table 1. Immunophenotyping of engrafted NSG (non-obese diabetic (NOD) evere combined immunodeficient (SCID) interleukin-2 receptor gamma null) (NOD.Cg-Prkdcscid Il2rgtm1Wjl/SzJ) mice was performed using frozen marrows from historical experiments (18–20 weeks post-transplant with 5,000 CB CD34^+^CD38^−^ HSPCs) as previously described [9].

**Table 1:** Information on the antibodies used in this study

Name	Source	Clone or reference	Volume or concentration/test
CD34 FITC	BD Biosciences	581	5 μL
CD34 PE	BD Biosciences	581	5 μL
CD34 PerCp	BD Biosciences	8G12	5 μL
CD34 APC	BD Biosciences	581	5 μL
CD34 Alexa Fluor (AF) 647	Biolegend	581	5 μL
CD38 APC-eFluor (eF) 780	eBioscience	HIT2	5 μL
CD45RA PE-Cy7	eBioscience	HI100	5 μL
CD45 FITC	BD Biosciences	HI30	5 μL
CD45RA Brilliant Violet (BV)785	Biolegend	HI100	5 μL
CD49f FITC	BD Biosciences	GoH3	5 μL
CD49f PE	BD Biosciences	GoH3	5 μL
CD49f PE-Cy5	BD Biosciences	GoH3	5 μL
CD49f AF647	BD Biosciences	GoH3H	5 μL
CD90 APC	eBioscience	5E10	5 μL
CD90 BV605	Biolegend	5E10	5 μL
CD201 APC	Biolegend	RCR-401	2.5 μL
CD201 PE	Biolegend	RCR-401	2.5 μL
CD201 APC	Miltenyi Biotec.	REA337	2 μL
CD201 PE	Miltenyi Biotec.	REA337	2 μL
CD201 PE	BD Biosciences	RCR-252	5 μL
CD370 PE	Biolegend	8F9	5 μL
CD370 APC	Biolegend	8F9	5 μL
Lineage cocktail eF450	eBioscience	22-7775-72	5 μL
GPRC5C Biotin	Bio-Techne	577315	0.5 μg/mL
Streptavidin FITC	BD Biosciences	554060	0.25 μL
Streptavidin AF700	Invitrogen	S21383	0.125 μL
Streptavidin BV711	Biolegend	405249	0.5 μL
PE/APC Rat IgG1k isotype control (IC)	BD Biosciences	R3-34	lot dependent: same amount of the specific antibody
FITC/PE/PE-Cy5/AF674 Rat IgG2ak IC	BD Biosciences	R35-95	2 μL
PE/APC/BV605 Mouse IgG1k IC	BD Biosciences	MOPC-21	5 μL
APC-eFluor 780 Mouse IgG1k IC	eBioscience	P3.6.2.8.1	5 μL
PE-Cy7/BV785 Mouse IgG2bk IC	Biolegend	MOPC-173	5 μL
Biotin Mouse IgG2ak IC	Bio-Techne	20102	0.5 μg/mL
PE/APC REA Human IgG IC	Miltenyi Biotec.	REA293	2 μL

### Immunophenotyping Considerations

Each antigen-specific antibody titration was performed using the staining condition described above with CBMNCs (cord blood mononuclear cells) or CB eCD34^+^ cells, using the antibody amount or concentration recommended by each manufacturer as a reference followed by one staining condition with double the recommended antibody amount and two to three twofold serial antibody dilution conditions. All the analyses were performed using the same voltage/condition. The selected antibody amount/concentration was determined based on the condition that required the minimum antibody amount that yielded the same/similar mean fluorescent intensity (MFI) and positive events as the plateau condition. An example of this is illustrated in Supplementary Figure E1A. Information on the antibody panels can be seen in Table 2.

**Table 2:** Information on the antibody panels used in this study

Figure and panel #	Fixed Ab panel	Alternate Abs
Figure 1 Supplementary Figure E2B	CD38 APC-eF780 CD90 BV605 CD45RA BV785	CD34 FITC/CD49f PE or CD34 PerCp/CD49f FITC or CD34 PerCp/CD49f PE or CD34 FITC/CD49f PE-Cy5 or CD34 PerCp/CD49f AF647 or CD34 FITC/PE Rat IgG2ak IC or CD34 PerCp/respective Rat IgG2ak IC or CD34 FITC/respective Rat IgG2ak IC
Figure 2A	CD34 FITC CD38 APC-eF780 CD90 BV605 CD45RA BV785 CD49f PE	CD201 APC (RCR-401) or CD201 APC (REA337) or APC Rat IgG1k IC or APC REA Human IgG IC
Figures 2B, C, and D	CD34 FITC CD38 APC-eF780 CD90 BV605 CD45RA BV785 CD49f PE-Cy5	CD201 APC (RCR-401) or CD201 APC (REA337) or APC Rat IgG1k IC or APC REA Human IgG IC; CD201 PE (RCR-401) or CD201 PE (REA337) or PE Rat IgG1k IC or PE REA Human IgG IC
Figure 3A	Lin eF450 CD45 FITC CD34 PerCp CD38 APC-eF780 CD90 BV605 CD45RA BV785	CD201 PE (RCR-401) or CD201 PE (REA337) or PE Rat IgG1k IC or PE REA Human IgG IC
Figure 3B	CD45 FITC CD34 APC CD38 APC-eF780 CD90 BV605 CD45RA BV785 CD49f PE-Cy5	CD201 PE (RCR-401) or CD201 PE (REA337) or PE Rat IgG1k IC or PE REA Human IgG IC
Figure 3C	CD34 FITC CD38 APC-eF780 CD90 APC CD45RA PE-Cy7	CD201 PE (RCR-401) or CD201 PE (REA337) or PE Rat IgG1k IC or PE REA Human IgG IC
Figure 4 Supplementary Figure E3	CD34 FITC CD38 APC-eF780 CD90 BV605 CD45RA BV785 CD49f PE-Cy5	CD201 APC (RCR-401)/CD370 PE or CD201 APC (REA337)/CD370 PE or APC Rat IgG1k IC/PE Mouse IgG1k IC or APC REA Human IgG IC/PE Mouse IgG1k IC; CD201 PE (RCR-401)/CD370 APC or CD201 PE (REA337)/CD370 APC or PE Rat IgG1k IC/APC Mouse IgG1k IC or PE REA Human IgG IC/APC Mouse IgG1k IC
Figure 5A	CD34 FITC CD38 APC-eF780 CD90 BV605 CD45RA BV785 CD49f PE-Cy5	GPRC5C biotin-streptavidin BV711/CD201 PE (REA337)/CD370 APC or Biotin mouse IgG2ak IC-streptavidin BV711/PE REA human IgG IC/APC Mouse IgG1k IC
Figure 5B Supplementary Figure E4B	CD45 FITC CD34 APC CD38 APC-eF780 CD90 BV605 CD45RA BV785 CD49f PE-Cy5	CD201 PE (RCR-401)/GPRC5C biotin-streptavidin BV711 or PE rat IgG1k IC/biotin mouse IgG2ak IC-streptavidin BV711
Supplementary Figure E1A	CD34 FITC CD38 APC-eF780	CD45RA PE-CY7 CD90 BV605 or PE-Cy7 mouse IgG2bk IC/BV605 mouse IgG1k IC
Supplementary Figure E1B	CD34 PerCp CD38 APC-eF780 CD45RA BV785	CD90 BV605/CD49f PE/CD201 APC (REA337) or BV605 mouse IgG1k IC/PE rat IgG2ak /APC REA human IgG IC
Supplementary Figure E2A	CD38 APC-eF780	CD34 PerCp/CD49f FITC or CD34 PerCp/CD49f PE or CD34 FITC/CD49f PE-Cy5 or CD34 PerCp/CD49f AF647 or CD34 PE or CD34 AF647
Supplementary Figure E4A	CD34 PerCp CD38 APC-eF780 CD90 BV605 CD45RA BV785	GPRC5C biotin-streptavidin FITC/CD201 PE (REA337)/CD370 APC or Biotin mouse IgG2ak IC-streptavidin FITC/PE REA human IgG IC/APC mouse IgG1k IC
Supplementary Figure E5	CD34 FITC CD38 APC-eF780 CD90 BV605 CD45RA BV785 CD49f PE-Cy5	GPRC5C biotin-streptavidin BV711/CD201 PE (REA337)/CD370 APC/CD49f PE-CY5 or biotin mouse IgG2ak IC-streptavidin BV711/PE REA Human IgG IC/APC mouse IgG1k IC

To determine the fluorescence thresholds, we used a combination of fluorescence minus one (FMO) controls, where all other specific staining in the same tube minus the one(s) of interest but replaced with the appropriate isotype-matched control(s), and an internal antigen-negative/low and/or antigen-positive expressing subpopulation(s) as guidance. This was possible within a heterogeneous population like CD34^+^ cells where the different subpopulations have similar levels of autofluorescent signal. An example of the gating strategy is illustrated in the Supplementary Figure E1B. Briefly, gates were set up first to exclude nonviable cells, debris, and doublets. Generally, CD34^+^CD38^−^ fraction was gated on approximately the lowest 7%–10% for CD38 expression in the CD34^+^ population. The delineation of this cell fraction is well-established to contain HSPCs with the highest *in vivo* repopulating capacity and the continuing increase in CD38 expression with the gradual loss of *in vivo* repopulating potential [10,11]. From this, we gated the CD45RA^−^ fraction by plotting CD90 versus CD45RA expression and the other subpopulations of interest within the CD45RA^−^ cell fraction. Of note, variation can occur between experiments, even using pooled samples. Thus, we highly recommend using internal negative controls within each sample, such as an antigen-negative subpopulation(s). As an example, we used CD34^+^CD38^−^CD45RA^+^ as a negative/low-expressing cell population as a reference for CD90, CD201, and CD370 expression. In general, stringent gates were applied with at least 0.4 of a log_10_ set apart from each other. We demarked the different subpopulations based on CD90 versus CD49f expression (P1–P4) by gating the 20%–25% of the highest and the lowest CD49f expression within the CD34^+^CD38^−^CD45RA^−^CD90^+^ and CD34^+^CD38^−^CD45RA^−^CD90^−^ populations.

The settings used for the LSRFortessa were as follows: 488 nm blue laser (50 mV): 505 long-pass (LP), 530/30 band-pass (BP), and 685 LP 710/50 BP; 561 nm yellow-green laser (50 mV): 586/15 BP, 600 LP 610/20 BP, 685 LP 710/50 BP, and 750 LP 780/60 BP; 355 nm ultraviolet (UV) laser (20 mV): 450/50 BP, 505 LP 530/30 BP, and 635 LP 670/30 BP; 640 nm red laser (40 mV): 670/14 BP, 690 LP 730/45 BP, and 750 LP 780/60 BP; 405 nm violet laser (55 mV): 450/50 BP, 505 LP 525/50 BP, 600 LP 610/20 BP, 630 LP 670/30 BP, 685 LP 710/50 BP, and 755 LP 780/60 BP. All the flow-cytometry analyses were performed using the FlowJo V10.10 software (BD Biosciences).

### Activated Protein C Treatment

Briefly, 5–10 × 10^4^ eCD34^+^ HSPCs were incubated with activated protein C (aPC) (ThermoFisher) at the concentration of 1 μg/mL in 50 μL of staining buffer for 60 min at 4°C. Cells were then washed once in the staining buffer followed by immunophenotyping as described above.

### Statistical Analysis

GraphPad Prism was used for all statistical analyses. Unless otherwise indicated in the figure legend, mean ± standard deviation [SD] values are reported in the graphs. Statistical significance was determined using Student (two-tailed) paired or unpaired *t* tests.

## RESULTS

### Detection of Weak CD49f Expression on Human HSPCs Can Be Mediated by Fluorochrome Choice

**Figure 1:**
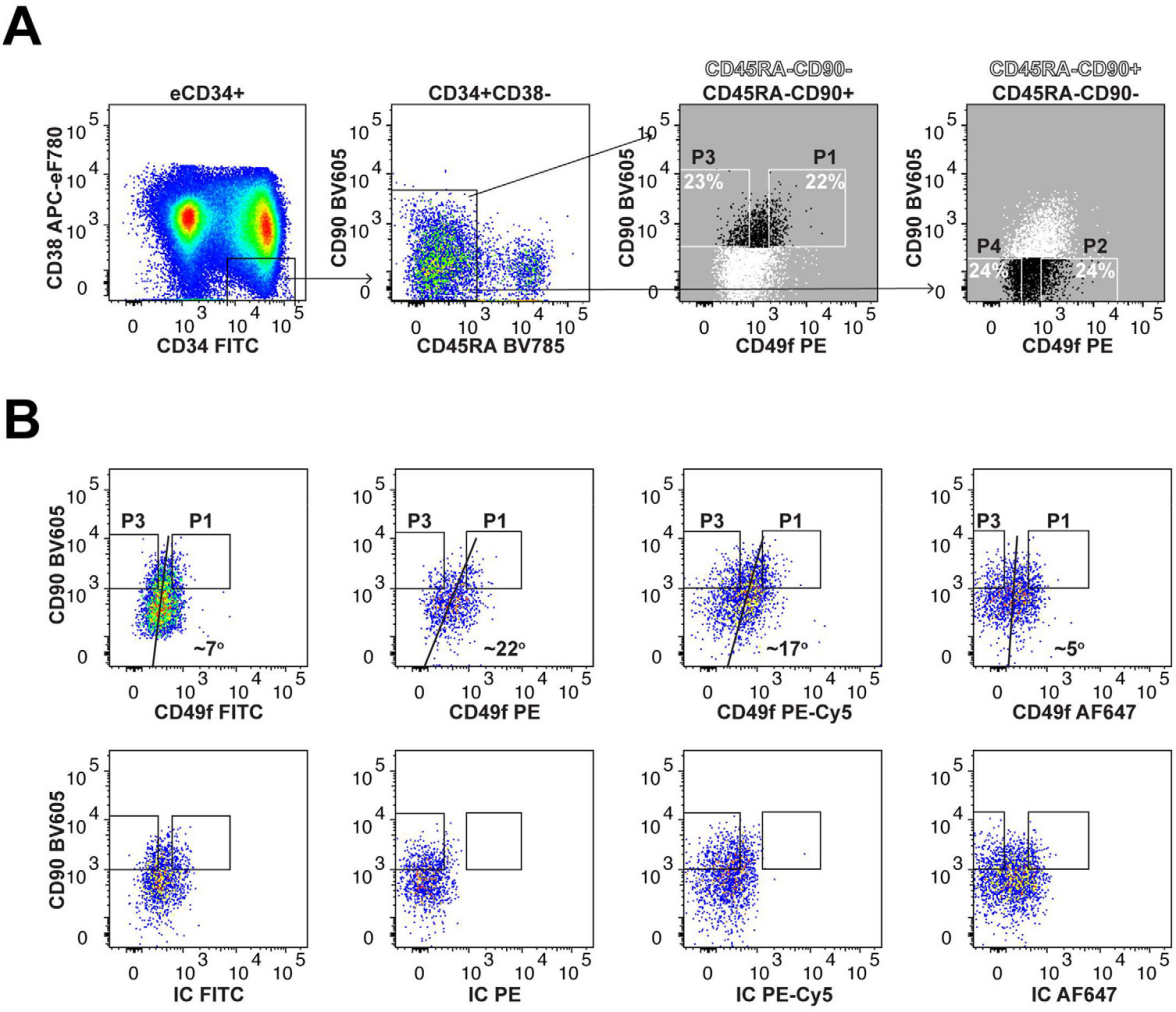
Outlining the gating strategy for human HSC enrichment and analysis of anti-CD49f antibody conjugates. **(A**) Representative flow-cytometry plots from multiple experiments depicting the gating strategy for CD34^+^CD38^−^CD45RA^−^ HSPCs enrichment and separation of the four subpopulations based on CD90 and CD49f expression: CD90^+^CD49f^+^ (P1), CD90^−^CD49f^+^ (P2), CD90^+^CD49f^−^ (P3) and CD90^−^CD49f^−^ (P4) (*n* = 7 independent pools of CB). The gates shown were based on the 20%–25% of the highest and the lowest CD49f expression within the CD34^+^CD38^−^CD45RA^−^CD90^+^ and CD34^+^CD38^−^CD45RA^−^CD90^−^ populations and not by the isotype controls (IC) stains. (**B**) Comparison of the different fluorochrome conjugates for the anti-CD49f antibody used. Representative pseudocolor plots using the indicated anti-CD49f antibodies (top plots) with their respective IC based on separate FMO stains (bottom plots) in CD34^+^CD38^−^CD45RA^−^ HSPCs. The same P1 and P3 gates were applied to the IC stains. The angle of distribution between positive and negative CD49f events was detected by the indicated anti-CD49f antibodies (*n* = 2 independent pools of CB).

Notta et al. [5] have effectively demonstrated that CD90 and CD49f as selection markers to enrich HSCs (eHSCs). Indeed, when combined with anti-CD34/CD38/CD45RA stains, four subpopulations of HSPCs can be defined in CB CD34^+^CD38^−^CD45RA^−^ cells. However, the definition of CD49f expression has been rather arbitrary, with the initial suggestion to delineate 25%–30% of the highest and the lowest CD49f expression on both CD34^+^CD38^−^CD45RA^−^CD90^+^ and CD34^+^CD38^−^CD45RA^−^CD90^−^ fractions [5]. As in our previous work, we used here a slightly more stringent approach by outlining 20%–25% of the highest and the lowest CD49f expression instead [6]. Thus, we refer to them here as P1 (CD90^+^CD49f^+^), P2 (CD90^−^CD49f^+^), P3 (CD90^+^CD49f^−^), and P4 (CD90^−^CD49f^−^) (Figure 1A).

The anti-CD49f antibody clone GoH3 has been the preferred choice. However, there remained poor resolution regarding the high or low CD49f expression on HSPCs (Figure 1). This was not due to the weak binding capacity of this antibody as it labeled well CD34^−^ cells from the partially eCD34^+^ cell preparation (Supplementary Figure E2A) or to the lower voltage applied, as using the same flow-cytometry settings bright signals were detected with the different conjugated anti-CD34 antibodies to stain the same cell preparation (Supplementary Figure E2A). The selection for the high and low CD49f expression events became even more problematic when certain fluorochromes were employed (Figure 1B, top plots). Fluorescein isothiocyanate (FITC)- or Alexa Fluor 647 (AF647)-conjugated anti-CD49f antibodies, exhibited very narrow separation between CD49f^+^ and CD49f^−^ cells when we outlined 20-25% of the highest and the lowest CD49f expression (Figure 1B, top plots). Indeed, when using the FMO staining method it was difficult to discern differences between primary antibody and isotype-matched control-stained cells (Figure 1B, top and bottom plots). Using phycoerythrin (PE) or phycoerythrin-cyanine5 (PE-Cy5) conjugated antibodies, positive and negative subpopulations were better highlighted (Figure 1B, top plots). Interestingly, the angle of distribution between positive and negative events was considerably higher when using PE or PE-Cy5 anti-CD49f as compared with FITC or AF647 anti-CD49f AF647 antibodies (Figure 1B, top plots), and the resulting “funnel-shaped” staining pattern was not due to compensation-related concerns (Supplementary Figure 2B). In addition, we observed a similar angle of distribution of the events when APC anti-CD90/PE anti-CD49f stains were performed (data not shown), where the spillover of the APC signal to the PE channel was unlikely. This was not observed in the APC anti-CD90/FITC anti-CD49f or PE anti-CD90/FITC anti-CD49f stains (data not shown), thus further supporting the importance of the brightness of the PE and PE-Cy5 anti-CD49f antibodies in highlighting the dim CD49f expression on human HSPCs. In summary, the choice of PE or PE-Cy5 fluorochromes for the anti-CD49f antibody is crucial when we wish to enrich for primitive CD49f^+^ HSPCs (P1 and P2) with fewer contaminants from the other two progenitor fractions (P3 and P4/MPP [multipotent progenitor]).

### PE-Conjugated Anti-CD201 Antibodies Enable Better Detection of Human EPCR^+^ HSCs

EPCR (CD201) was the main focus of this work. We have previously determined the *in vivo* repopulating cell capacity of EPCR^+^ HSCs as approximately one in three cells [6]. These results were reproducible with the 3 different antibody clones, including RCR-227 (eBioscience), RCR-401 (BioLegend), and RCR-252 (BD Biosciences), all of which were conjugated to APC. Importantly, little difference was seen between their capacity to highlight EPCR-positive events in CB CD34^+^CD38^−^CD45RA^−^ cells [6]. In our previous report, when we did not need to stain human CD34^+^ HSPCs for CD49f antigen (using the best-tested conjugated antibody, PE), we exchanged APC for PE anti-CD201 antibodies. We then observed that the PE-conjugated RCR-401 and RCR-252 antibodies gave a brighter signal and therefore slightly more EPCR^+^ events than their respective APC-conjugated ones [6]. Of note, a PE conjugate for RCR-227 was and remains not commercially available to be tested. However, in later experiments, the efficacy of the PE anti-CD201 RCR-252 antibody was found to be lot dependent. In some lots, the antibody was unable to detect any EPCR^+^ cells in CD34^+^ HSPCs, whereas this was not the case for the other antibody clone (data not shown). As a result of this pitfall, we decided to test and report here the usefulness of an additional anti-CD201 antibody, clone REA337 (Miltenyi Biotec) and used the RCR-401 antibodies as a reference.

**Figure 2:**
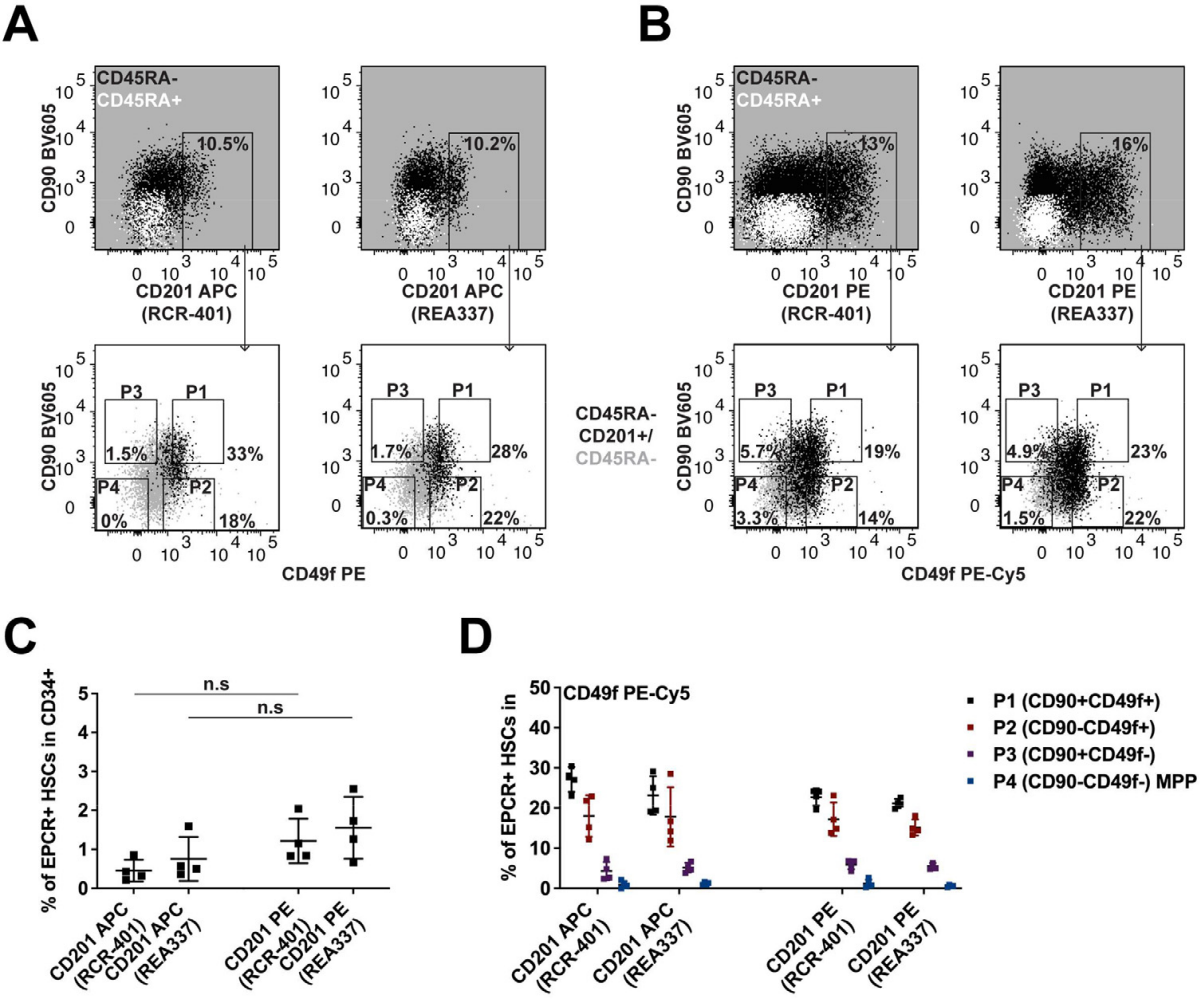
Detection of EPCR expression and its coexpression with integrin-α6 on human HSPCs. Representative overlay flow-cytometry plots illustrating (**A**) APC anti-CD201 and PE anti-CD49f or (**B**) PE anti-CD201 and PE-Cy5 anti-CD49f stains with either RCR-401 or REA337 anti-CD201 antibodies in CD34^+^CD38^−^CD45RA^−or+^ cells. Gating is shown against the four CD90/CD49f populations (see Material and Methods for explanation) The remaining gates were based on the combination of IC stains and using CD34^+^CD38^−^CD45RA^+^ LMPP/MLP cells as an internal negative control for EPCR expression (top plots; overlay white dots). CD34^+^CD38^−^CD45RA^−^ cells (bottom plots; underlay grey dots) were used to illustrate the overall CD49f staining pattern. (**C**) Percentage of EPCR^+^ cells in the CD34^+^ population using the depicted antibody clones and their respective conjugates (*n* = 4 independent pools of CB). **(D**) Distribution of EPCR^+^ HSCs within P1, P2, P3, or P4 fractions (*n* = 4 independent pools of CB). The frequencies were calculated based on the gates shown. n.s.=Nonsignificant.

As previously observed, the PE anti-CD201 RCR-401 antibody gave a slightly greater detection of EPCR^+^ events (although not statistically significant) compared with the APC-conjugated version (Figure 2A–C). This was also the case for the anti-CD201 REA337 antibodies (Figure 2A–C). Of note, apart from one odd CB experiment, in most of the pool of samples used in this and other studies (data not shown), the frequencies of EPCR^+^ HSCs detected were still low (usually ∼0.6 to ∼1% of CB CD34^+^ cells) even when using a PE anti-CD201 antibody to detect EPCR expression (Figure 2C). Importantly, EPCR^+^ HSCs were mostly found within the P1 and P2 populations when using the PE anti-CD49f antibody in combination with an APC anti-CD201 antibody [6]. Repeating this work, we were able to confirm this and observed that the APC anti-CD201 REA337 antibody gave similar results as the RCR-401 antibody (Figure 2A). When using a PE anti-CD201 antibody in combination with the PE-Cy5 anti-CD49f antibody this finding was also confirmed, with the greatest percentage of EPCR^+^ cells existing within the P1 fraction, followed by P2, with P3 and P4 containing far fewer EPCR^+^ cells (Figure 2B). However, due to the slightly dimmer nature of the fluorochrome PE-Cy5 as compared with PE, these were slightly higher percentages in P3 and P4 than those detected with the PE anti-CD49f antibody (Figure 2A, B). Overall, these experiments suggest that a PE-conjugated anti-CD201 antibody should be the preferred option for EPCR^+^ HSC detection. In addition, the REA337 antibodies performed as efficiently as the RCR-401 antibodies not only in detecting EPCR^+^ cells in CB CD34^+^ HSPCs but also in giving a very comparable distribution pattern within the different HSPC subpopulations delineated by CD90 and CD49f expression (Figure 2D). Indeed, as expected, we did see differences in staining index between PE versus APC anti-CD201 antibodies but not between the two clones (PE anti-CD201 RCR-401 antibody with a staining index of 7.01 ± 0.98 and PE anti-CD201 REA337 with 7.07 ± 1.39; APC anti-CD201 RCR-401 with 3.78 ± 0.84 and APC anti-CD201 REA337 with 4.1 ± 0.57).

### EPCR Detection Varies With Tissue Sources and Antibody Clones

**Figure 3:**
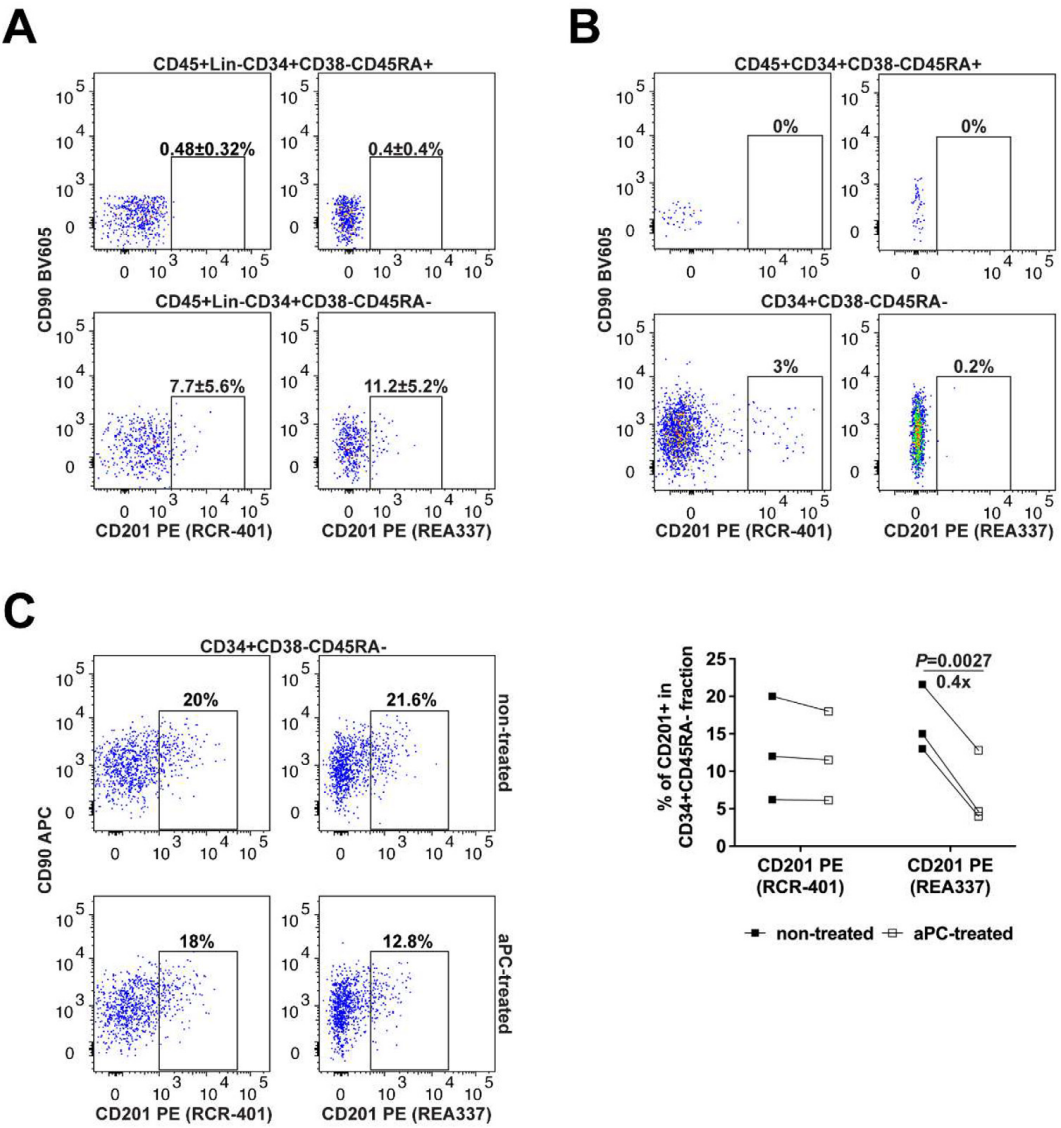
Comparison of anti-CD201 antibody clones for EPCR^+^ HSC detection in different cellular sources (**A**) Detection of EPCR^+^ cells in human CD45^+^Lin^−^CD34^+^CD38^−^CD45RA^−^ HSPCs using RCR-401 or REA337 anti-CD201 antibodies in the marrows of NSG mice (18–20 weeks post-transplant). ± shown is the SD for the number of experiments performed (*n* = 4 individual mice). CD45^+^Lin^−^CD34^+^CD38^−^CD45RA^+^ LMPPs/MLPs within each sample/stain were used as an internal negative control for EPCR expression. (**B**) Detection of EPCR^+^ cells in human BM-derived (BM1) CD34^+^CD38^−^CD45RA^−^ HSPCs. CD34^+^CD38^−^CD45RA^+^ LMPPs/MLPs were used as an internal negative control for EPCR expression. (**C**) Representative flow-cytometry plots illustrating the expression of EPCR detected on nontreated and aPC-treated CB eCD34^+^ HSPCs using the anti-CD201 RCR-401 or REA337 antibodies. The graph represents the quantification of the experiments performed (*n* = 3 independent pools of CB).

The antibody clones used and discussed here highlighted EPCR^+^ cells in CB CD34^+^ HSPCs well, as well as in xenografts for functional assays even at 18–20 weeks post-transplant. Comparing RCR-401 and REA337 antibodies for EPCR detection of NSG-engrafted cells, there was little difference between the two antibody clones, with the anti-CD201 REA337 antibody being marginally better (Figure 3A). There were however more differences observed with BM-derived eCD34^+^ HSPCs. Anti-CD201 REA337 antibody imparts very poor detection of EPCR (Figure 3B) as compared with the anti-CD201 RCR-401 antibody. According to the anti-CD201 REA337 antibody manufacturer's information, this antibody seems to bind an epitope shared by the anti-CD201 RCR-401 antibody. We hypothesize however that the epitopes detected by the two clones do not entirely overlap and the epitope detected by the anti-CD201 REA337 antibody could be blocked in the presence of certain EPCR substrates such as aPC. To test this, we incubated CB eCD34^+^ HSPCs with aPC and then stained them for EPCR using either of the two antibodies. aPC treatment led to a significant decrease in EPCR detection by ∼60% (in terms of percentages of CD201^+^ events and not in MFI) only when the anti-CD201 REA337 but not the anti-CD201 RCR-401 antibody was used (Figure 3C). These results suggest that the detection of certain CD201 epitopes on BM-derived EPCR^+^ HSCs was impaired when using the anti-CD201 REA337 antibody, likely due to being blocked, at least in part, by the aPC present in the BM microenvironment.

### CLEC9A Expression is Unlikely to Further Refine EPCR^+^ HSC Purity

Before the work identifying EPCR^+^ HSCs, other studies had trialed alternative markers to better isolate the most primitive CD34^+^ cells, including CLEC9A (or CD370). CLEC9A is a type II transmembrane receptor with a c-type lectin domain and an intracellular tyrosine activation region [10]. It was previously thought to only be expressed on a subpopulation of dendritic cells but has since been shown to be expressed on some monocytes and B cells and may induce the production of inflammatory cytokines [12,13]. More recently, it was reported to be highly expressed on primitive CD34^+^CD38^−^CD45RA^−^CD90^+^CD49f^+^ cells [7]. Therefore, we hypothesized that it may be possible to subfractionate ECPR^+^ HSCs with CLEC9A to further improve HSC enrichment capacity.

**Figure 4:**
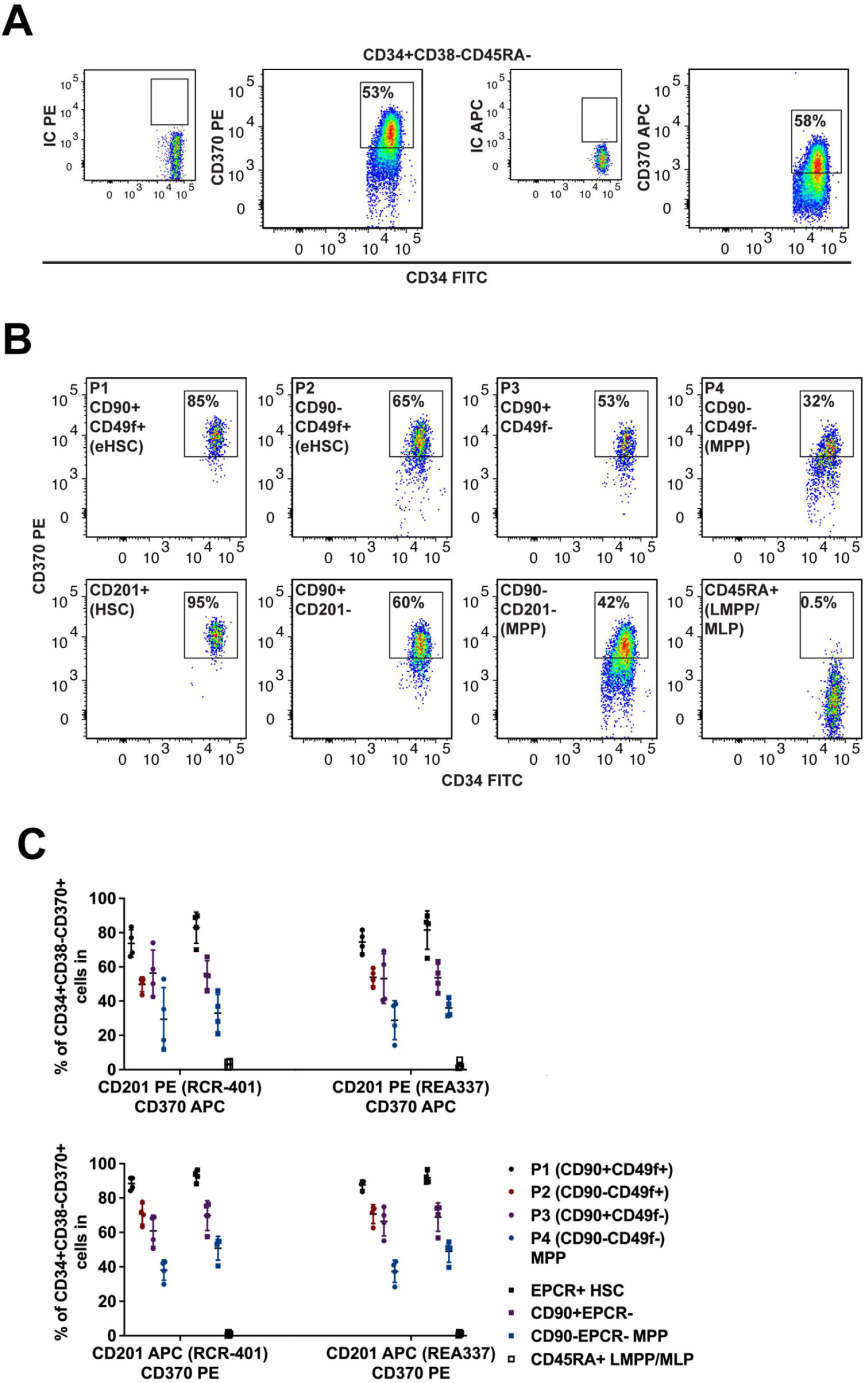
Defining the expression pattern of CLEC9A on human CB HSPCs. **(A**) Comparison of PE and APC as conjugates for anti-CD307 antibody stains in CD34^+^CD38^−^CD45RA^−^ HSPCs, alongside IC in separate stains (using the FMO method). (**B**) Representative flow-cytometry plots illustrating the expression of CLEC9A in the indicated populations using PE anti-CD307/PE-Cy5 CD49f/APC anti-CD201 antibodies. (**C**) Percentage of CD34^+^CD38^−^CD370^+^ cells in the denoted HSPC subpopulations defined by CD90/CD49f or CD90/EPCR expression using different antibody combinations (*n* = 4 independent pools of CB). The frequencies were calculated based on the gates shown. eHSCs=Enriched hematopoietic stem cells.

With the aim of first reproducing previous results, CLEC9A^+^ cells were found to include many CD34^+^CD38^−^CD45RA^−^ HSPCs, having been tested with both PE- and APC-conjugated variants as previously carried out with the antibody clone 8F9 (Figure 4) [7]. As with the anti-CD49f and anti-CD201 antibodies, the PE anti-CD370 antibody enabled a brighter signal than the APC conjugate (Figure 4A). The expression of CLEC9A was confirmed in different CB HSPC populations: P1 to P4, as well as EPCR^+^ HSCs, CD90^+^EPCR^−^ HSPCs, CD90^−^EPCR^−^ MPPs and CD45RA^+^ lymphoid-primed multipotent progenitors (LMPPs)/multipotent lymphoid progenitors (MLPs) (Figure 4B). In the uncovering of CLEC9A as a potential marker, Laurenti's group used a PE conjugate to detect CLEC9A, which has also been shown to be a better option for EPCR here [7]. Subsequently, combinations were explored: either APC anti-CD201 and PE anti-CD370, or vice versa, as well as testing both RCR-401 and REA337 clones of anti-CD201 antibodies as before (Figure 4C). Regarding P1 to P4 populations, expression of CLEC9A in CB was greatest in P1, with 83.6%–91.5% of P1 cells being CLEC9A^+^, when using PE anti-CD370 combined with PE-Cy5 anti-CD49f and either of the APC anti-CD201 antibodies. This dropped to 62.6%–74.4% for P2, 50.6%–74.8% for P3 and 28.4%–42.8% for P4, thus confirming previous work [7]. When explored in the context of EPCR^+^ HSCs, 88.2%–96.6% of these cells were also CLEC9A^+^, moving to 56.6%–76.3% of CD90^+^EPCR^−^ cells and 39.7%–54.8% of CD90^−^EPCR^−^ MPPs. In the case of the LMPP/MLP fraction, as few as 0.24%–2% of cells were CLEC9A^+^ (Figure 4C). As expected, the frequencies of CLEC9A^+^ cells in each of the HSPC subpopulations were slightly lower when APC anti-CD370 antibodies were used due to the dimmer nature of APC as compared with PE (Figure 4C). Nevertheless, >82% of EPCR^+^ HSCs were also positive for CLEC9A expression (Figure 4C). CLEC9A seems to be highly expressed on most EPCR^+^ HSCs, in particular when PE anti-CD370 antibodies were used (>90%); a few EPCR^+^ HSCs had a slightly lower level of CLEC9A expression, which fell just under our gates although these were not truly negative events. Therefore, our data suggest the use of CLEC9A being less justifiable as an additional selection marker for these HSCs; however, it may be of use to delineate distinct subpopulations within other progenitor populations where a more distinctive negative CLEC9A subfraction can be detected. Indeed, when selecting the few CD370^−^ cells within the P1 population most of them were CD90^+^EPCR^−^ progenitors [6] (Supplementary Figure E3). These results were consistent with previous findings showing that both CB CD34^+/lo^CD38^−^CD45RA^−^CD90^+^CD49f^+^CD370^−^ cells [7], as well as CD34^+^CD38^−^CD45RA^−^CD90^+^EPCR^−^ [6], were lymphoid-primed progenitors.

### GPRC5C is Not Useful for Primitive CD34^+^ HSC Enrichment

An additional cell surface antigen that has been explored for HSC isolation is GPRC5C, a G protein-coupled receptor characterized by a seven-transmembrane domain motif. Previously, GPRC5C expression had been shown to correlate with quiescence within the mouse HSC compartment, with the enrichment for dormancy corresponding to hyaluronic acid signaling [8]. Subsequently, we predicted that this antigen may be used alongside EPCR to better isolate primitive CD34^+^ HSCs. As before, results published previously were attempted to be validated before assessing the ability to better enhance the isolation of HSCs in combination with EPCR. The clone used in our work (577315, Bio-Techne) was the same used in the original report [8], biotinylated then conjugated with either of the three fluorochromes, two of which were also used for their work.

**Figure 5:**
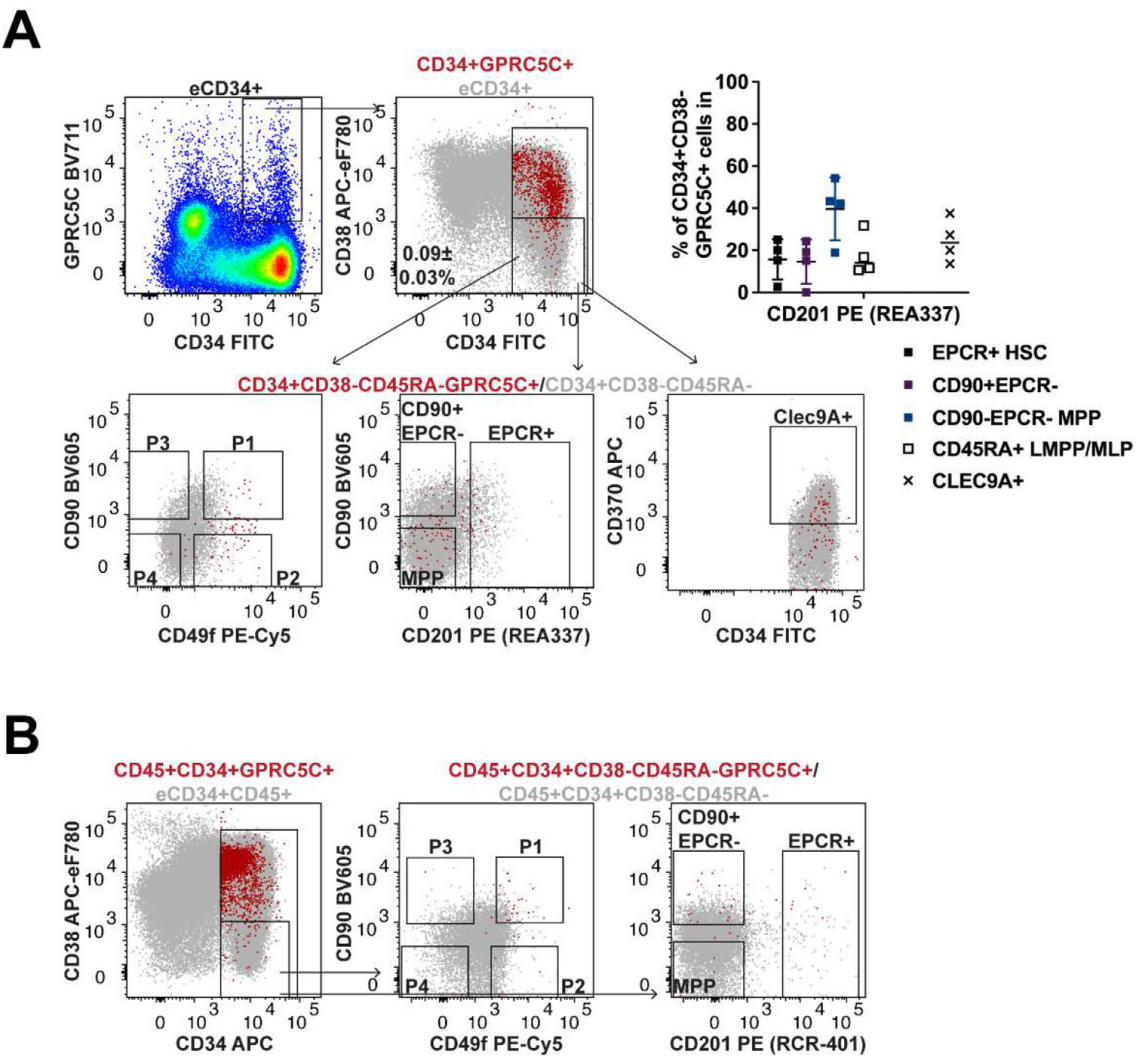
GPRC5C is mostly expressed on human CD38^+tohi^ progenitors. (**A**) Representative flow-cytometry plots denoting the selection of CB-derived CD34^+^GPRC5C^+^ cells and their back-gating onto the indicated populations. The inset graph represents the quantification of the experiments performed (*n* = 4). The frequencies were calculated based on the gates shown. (**B**) Representative flow-cytometry plots exemplifying BM-derived CD34^+^GPRC5C^+^ cells (red dots; BM1) and their back-gating onto the designated populations (*n* = 2 independent BM). eCD34^+^ or CD34^+^CD38^−^CD45RA^−^ cells (underlay grey dots) are shown to illustrate the overall staining pattern.

As reported, the expression of GPRC5C on CB CD34^+^ HSPCs had a “smear” like pattern without a well-defined subpopulation [8]. Importantly, when CD34^+^GPRC5C^+^ events were backgated, it was clear that most of them were in fact CD38^+^ to CD38^hi^ cells (Figure 5A). Furthermore, when selecting the very few GPRC5C^+^ cells that were phenotypically CD34^+^CD38^−^, they fell into different fractions with most being in the P2 fraction (Figure 5A). Despite being CD49f expressing cells, these were MPPs as they were CD90^−^EPCR^−^CD45RA^−^. In addition, only ∼25% of these CD34^+^CD38^−^CD45RA^−^GPRC5C^+^ cells were CLEC9A^+^ cells. These results suggest that most CB CD34^+^GPRC5C^+^ cells were not phenotypically classified as HSCs. We also attempted to use the anti-GPRC5C antibody conjugated with FITC with the hope of enhancing the detection of GPRC5C-positive events [8]. This also failed to improve the outcomes with most of the CB CD34^+^GPRC5C^+^ events detected being CD38^+^ to CD38^hi^ as before (Supplementary Figure E4A). We also performed the same stains using AF700 as in the original paper [8], which resulted in the same pattern (data not shown).

In the event the difference in staining might be related to differences between cellular sources, experiments were also performed using BM-derived HSPCs. As in CB, most BM-derived CD34^+^GPRC5C^+^ cells were CD38^+^ to CD38^hi^ cells. In the two BM samples tested, the few CD34^+^CD38^−^CD45RA^−^GPRC5C^+^ cells were phenotypically CD90^+^CD49f^+^ (P1) but many were not EPCR^+^ (Figure 5B). Out of interest, we ran a separate analysis by substantially increasing the gating for CD38 expression as previously shown [8]. Using this approach an approximately fourfold increase in the frequency of CD34^+^CD38^−/+tohi^CD45RA^−^CD90^+^CD49f^+^ (P1) cells was detected in the CD34^+^GPRC5C^+^ compared with the CD34^+^GPRC5C^−^ fraction (Supplementary Figure E4B). This was comparable with the previous work where an approximately threefold enrichment of P1 cells was obtained based on similar calculations and comparisons [8]. Overall, several experiments were carried out using both CB-derived and BM-derived HSPCs, and it was possible to reproduce previous data with most of the CD34^+^GPRC5C^+^ cells being CD38^+^ to CD38^hi^ but most of them were EPCR^−^. Thus, this eliminates the potential for GPRC5C to be used in combination with ECPR as intended.

At last, to probe the potential variation of the different HSPCs described, we stained individual CB samples using one of the best antibody combinations (Supplementary Figure E5). As expected, we observed minor variations between the samples, with frequencies of EPCR^+^ HSCs varying from 0.3%–1% of CD34^+^ cells.

## DISCUSSION

This work aimed to evaluate the methodology for CD34^+^ HSC isolation, exploring differences in antibody clones, conjugates, cell source, and additional cell surface markers for enhancing the purity of these cells. First, as discussed in the recent review, the integrin integrin-α6 (CD49f) is weakly expressed on HSPCs, making the detection of it challenging [14]. In addition, the fluorochrome of choice for the anti-CD49f antibody also plays a crucial role in how we can define 20%–25% of the highest CD49f expression on CD34^+^CD38^−^CD45RA^−^ HSPCs with fewer contaminants from the two progenitor fractions (P3 and P4/MPP). Indeed, a poor delineation of CD49f expression can alter the biological interpretation of experiments because we have previously shown that P1 cells sorted using AF647 anti-CD49f antibodies (without a “funnel-shaped” staining pattern) mostly failed to engraft at limiting cell doses as compared with cells sorted using PE anti-CD49f antibodies (with a “funnel-shaped” staining profile). This was likely due to the presence of many P3 progenitor cells that were shown to have a repopulating frequency of ∼1 in 90 cells [6]. Nevertheless, when using the PE anti-CD49f antibodies we were able to reproduce previous results with P1 having an *in vivo* repopulating frequency of ∼1 in 8.3 cells [5,6].

Subsequently, we searched for additional cell surface antigens that had been published around the time of the works on EPCR [6,15] to enhance the isolation of HSCs to a greater level. Two potential cell surface antigens of interest were explored: CLEC9A and GPRC5C. The work on CLEC9A showed within the P1 population a continuum of expression opposite to that of CD34, whereby CD34^+/hi^CLEC9A^−^ cells were shown to be lymphoid-primed and CD34^+/lo^CLEC9A^+^ cells to be multipotent [7]. Therefore, this appeared to be a useful marker to test as EPCR^+^ HSCs have also been demonstrated to be multipotent [6]. Here, we confirmed that CLEC9A was well expressed in P1 (CD90^+^CD49f^+^) eHSCs but importantly even more so on EPCR^+^ HSCs. Nevertheless, we felt that this additional marker may not be useful to further define EPCR^+^ HSCs due to the vast majority of EPCR^+^ HSCs being CLEC9A^+^. Moving on to GPRC5C, it was possible to detect GPRC5C expression on CD34^+^ HSPCs but most of the CD34^+^GPRC5C^+^ were CD38^+^ to CD38^hi^ cells. On carefully examining their data [8], this was not necessarily an unusual finding. As shown in the original gating strategy, most of the cells that were selected when analyzing CD34^+^GPRC5C^+^ cells were CD38^+tohi^. The method used to select CD34^+^CD38^−/lo^ cells was in our opinion not the most appropriate. We would not consider these cells to be CD38^−/lo^, but CD38^−to+/hi^. Many CD38^+^ cells are also known to express CD49f and CD90, which would indeed point toward a primitive HSPC phenotype. Although we did not present any functional data in this study, based on the results presented here, we believe that the CD34^+^CD38^−^CD45RA^−^EPCR^+^ phenotype continues to provide the simplest and highest level of enrichment of human HSCs, as we have shown that this represents the most purified human HSC population to date based on multiple functional studies previously reported [6].

At last, and most importantly, there were minimal differences observed between the two anti-CD201 antibody clones in most of the assays used, except in detecting BM EPCR^+^ cells. This may arise from differences with target epitopes. CB- and BM-derived HSCs naturally exist within different environments, and the combinations of adjacent cell surface proteins, for example, the proximity of EPCR to PAR1 for PAR1-thrombin signaling, may make certain epitopes less accessible. Indeed, when CB cells were treated with aPC we observed a significant reduction in the ability of the anti-CD201 REA337 antibody to detect EPCR expression.

For most antibodies against antigens that enrich stem cells (integrin-α6, EPCR, and CLEC9A), PE was found to be better for cell detection, in comparison to APC for instance. As a compromise, it was determined that the best combination would be BV605 for CD90, PE-Cy5 for CD49f, PE for EPCR, and APC for CLEC9A (or vice versa for the latter two), if all these stains are required to be used simultaneously. In this study, we purposely decided not to use any of the aforementioned antibodies in PE-Cy7 format to avoid potential spillover to the PE and/or PE-Cy5 channels to support our view that PE and PE-Cy5 were better fluorochrome conjugates as compared with FITC and AF647 for the anti-CD49f antibodies (giving the “funnel-shaped”) to highlight the dim CD49f expression on human HSPCs. We observed that PE-Cy7, PE and APC anti-CD90 antibodies of the same antibody clone (data not shown and Figure 3C) were better at highlighting CD90^+^ events, and these antibodies could be used instead. As such, appropriate antibody panels are essential to properly highlight rare cell populations, particularly when these cells have a dim expression of certain surface antigens (e.g., CD49f and CD90). Undoubtedly, certain compromises are required to be made when multicolor stains are needed. Although we made a few compromises (e.g., using BV605 anti-CD90 antibody), we believe that this was the best antibody combination possible in a multicolor stain and analyzed in a standard five-laser flow cytometer. With the advent of new flow cytometers, such as spectral flow cytometers, these concerns can potentially be overcome. In addition, we also suggest when using APC anti-CD201 antibodies for cell sorting to apply a more stringent gating approach by delineating the CD90^+^CD201^−^ gate well apart from the CD201^+^ gate to avoid possible contamination from the CD201^lo^ expressing cells. That said, if researchers prefer to isolate only EPCR^+^ HSCs and not the other progenitors, hence the quantity of the former is an important factor, we suggest using a simple stain with anti-CD34/CD38/CD45RA/CD201 antibodies, where a PE anti-CD201 antibody is used.

In the case of anti-CD201 antibody clones, lot differences have been noticed with the RCR-252 clone. Two to three lots were also trialed for REA337 and RCR-401, but fortunately, no differences have been found so far. However, we do recommend testing each individual antibody lot. Overall, we have highlighted the importance of experimental planning and antibody panel selection concerning the isolation of HSCs from multiple sources, which should enable better reproducibility of results between laboratory tests, as well as to further pursue improving the enrichment of human HSCs.

## Conflicts of Interest Disclosure

The authors do not have any conflicts of interest to declare in relation to this work.

## Appendix Supplementary materials

Image, application 1

## Footnotes

Supplementary material associated with this article can be found in the online version at https://doi.org/10.1016/j.exphem.2025.104729.
