# Supplementary material for: Methodologic considerations on how to identify human hematopoietic stem cells
Source: Exp Hematol. 2025 Apr;144:None. doi: 10.1016/j.exphem.2025.104729 (PMC13171471; doi:10.1016/j.exphem.2025.104729)
Supplement: Supplementary file 1 [file mmc1.docx]

**Supplementary figures and legends**

**Methodological Considerations on How to Identify Human Hematopoietic Stem Cells**

Taylor Hinchly, Dominique Bonnet and Fernando Anjos-Afonso.


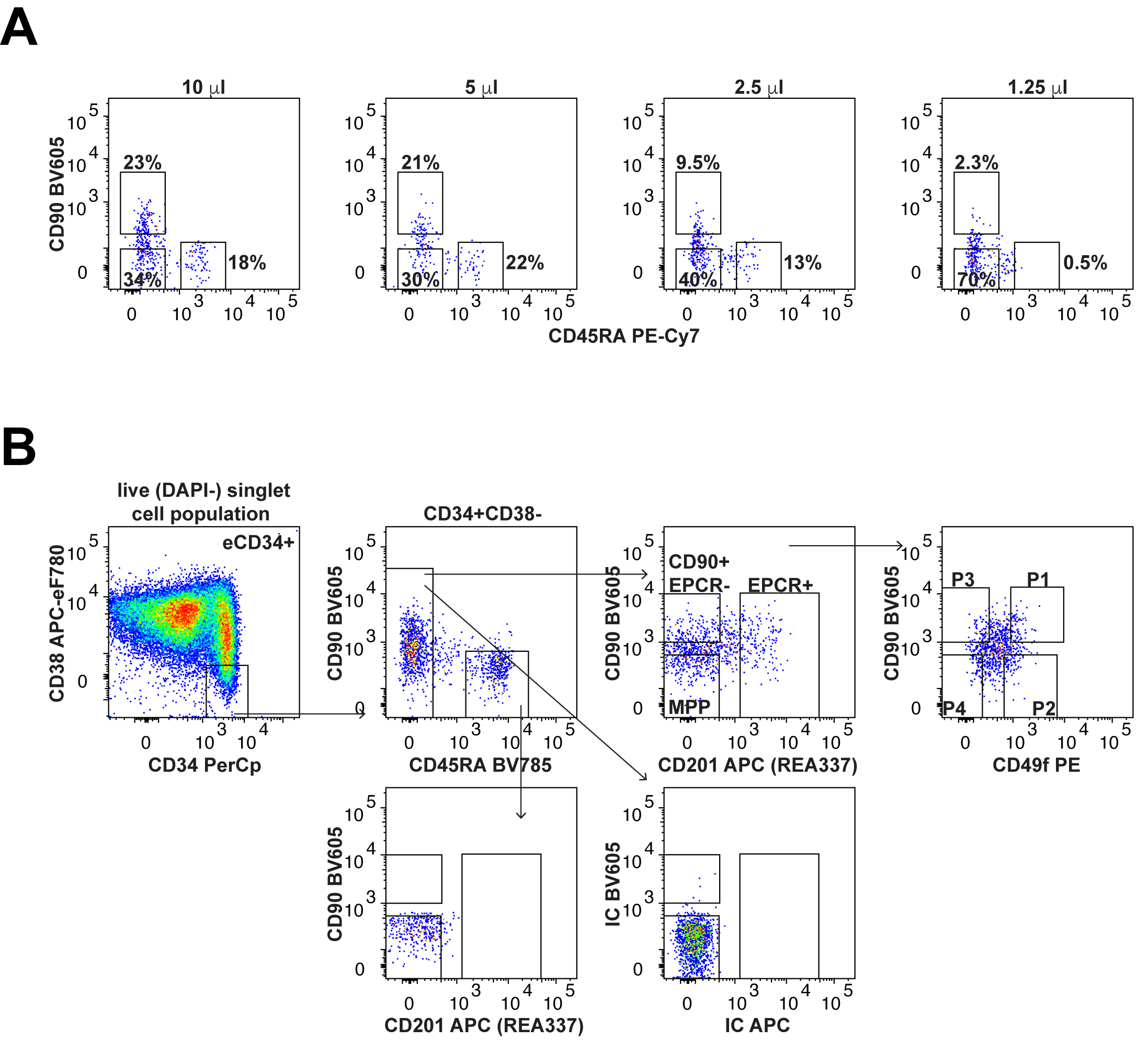


**Supplementary Figure E1.**

**A**) Representative flow-cytometry plots depicting the detection of CD90 and CD45RA expression on CB CD34^+^CD38^-^ cells with the indicated amount of BV605 anti-CD90 and PE-Cy7 anti-CD45RA antibodies to stain the same cell preparation. The recommended amount for these antibodies by their respective manufacturer was 5 μl/stain. As shown, with 5 μl, the M.F.I. of CD90/CD45RA expression and the percentages of positive events were comparable to the 10 μl condition, contrary to the other conditions where fewer antibodies were used. It was then determined that 5 μl/stain was the optimum amount for these antibodies. **B**) Representative flow-cytometry plots illustrating the generic gating strategy used to define the indicated sub-populations within CD34^+^CD38^-^ HSPCs. CB eCD34^+^ cells were used as an example. In this case, the CD34^+^CD38^-^ fraction was gated on the lowest 10% for CD38 expression in the CD34^+^ population. As a suggestion, the CD34^-^CD38^+^ sub-fraction within eCD34^+^ cells was able to aid in delineating the CD34^+^CD38^-^ fraction. Within the CD34^+^CD38^-^CD45RA^-^ fraction, it was possible to outline the indicated sub-populations based on CD90 versus EPCR (CD201) or CD90 versus CD49f expression. The gates were determined based on the combination of isotype controls using the F.M.O. method and also using the CD34^+^CD38^-^CD45RA^+^ population as an internal reference for negative/low CD90 and CD201 expression. P1 to P4 gates were gated based on the 20-25% of the highest and the lowest CD49f expression within the CD34^+^CD38^-^CD45RA^-^CD90^+^ and CD34^+^CD38^-^CD45RA^-^CD90^-^ populations.


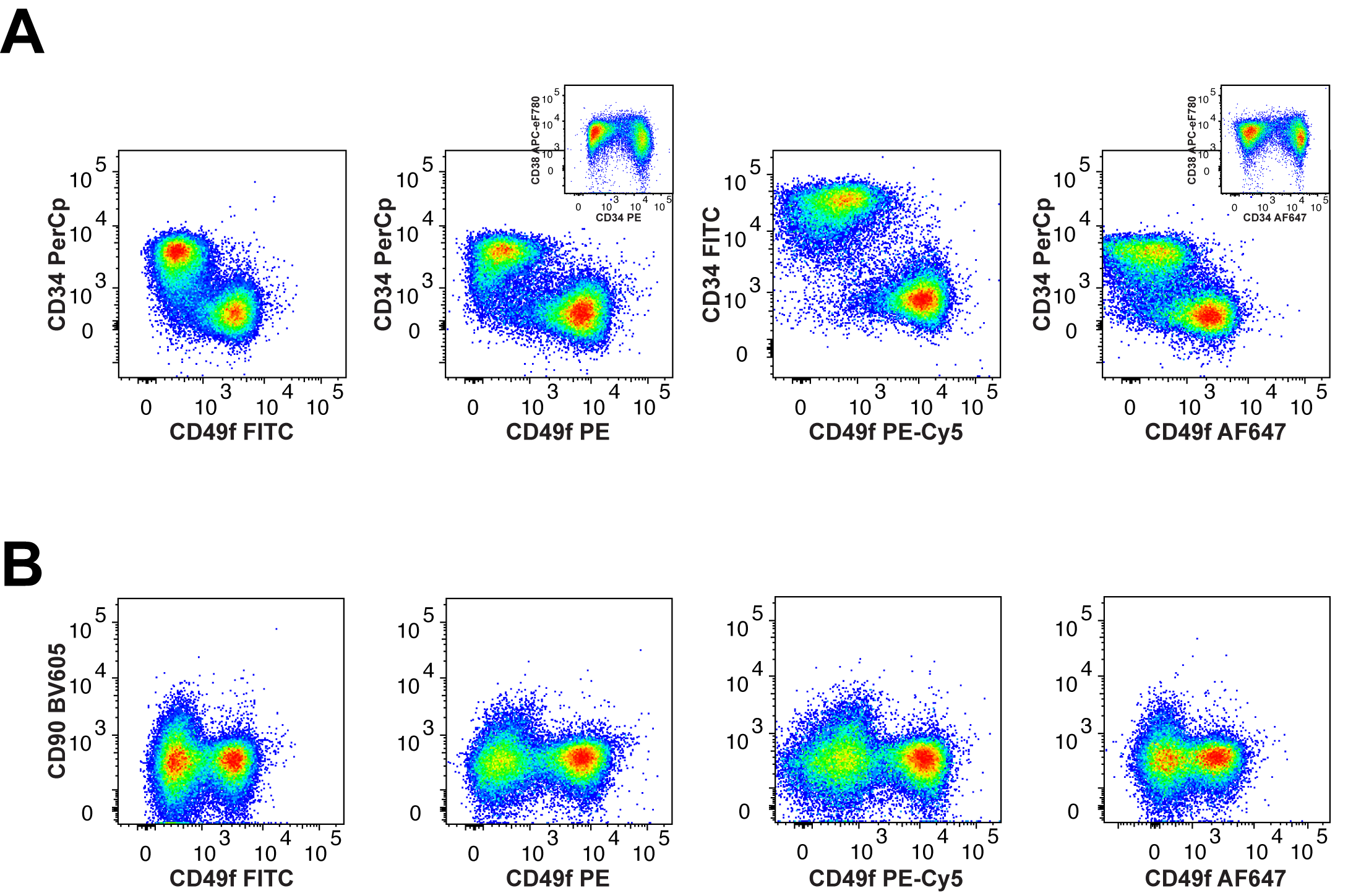


**Supplementary Figure E2.**

**A**) Representative flow-cytometry plots depicting the detection of CD34 and CD49f expression on human CB eCD34^+^ cells with the different conjugated anti-CD34 and anti-CD49f antibodies to stain the same cell preparation using the same flow-cytometry voltage settings for each fluorochrome. **B**) Representative flow-cytometry plots illustrating the appropriate compensation between the denoted anti-CD49f with BV605 anti-CD90 antibody stains.


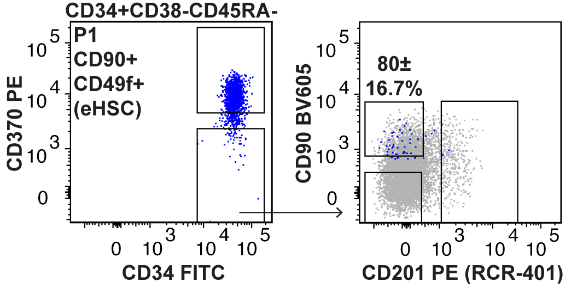


**Supplementary Figure E3.**

Representative flow-cytometry plots exemplifying CB-derived CLEC9A^-^ cells within P1 fraction (CD34^+^CD38^-^CD45RA^-^CD90^+^CD49f^+^) and their back-gating onto the designated populations. CD34^+^CD38^-^CD45RA^-^ cells (underlay grey dots) are shown to illustrate the overall staining pattern. ± shown is the S.D. for the no. of experiments performed (n=4 independent pools of CB).


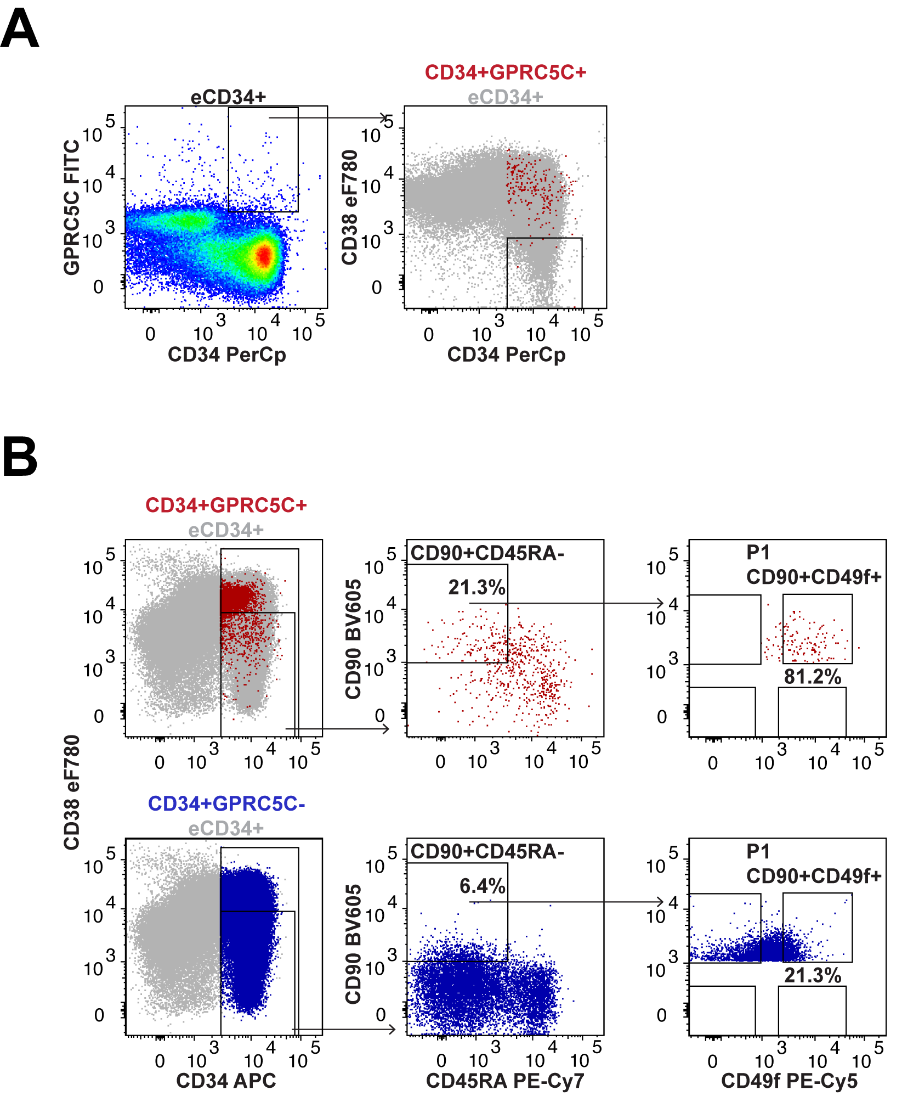


**Supplementary Figure E4.**

**A**) Representative flow-cytometry plots denoting the selection of CB-derived CD34^+^GPRC5C^+^ cells and their back-gating onto the indicated populations. **B**) Representative flow-cytometry plots exemplifying BM-derived CD34^+^GPRC5C^+^ (top plots; red dots; BM1) or CD34^+^GPRC5C^-^ (bottom plots; blue dots) cells and their back-gating onto the designated populations. eCD34^+^ or CD34^+^CD38^-^CD45RA^-^ cells (underlay grey dots) are shown to illustrate the overall staining pattern.


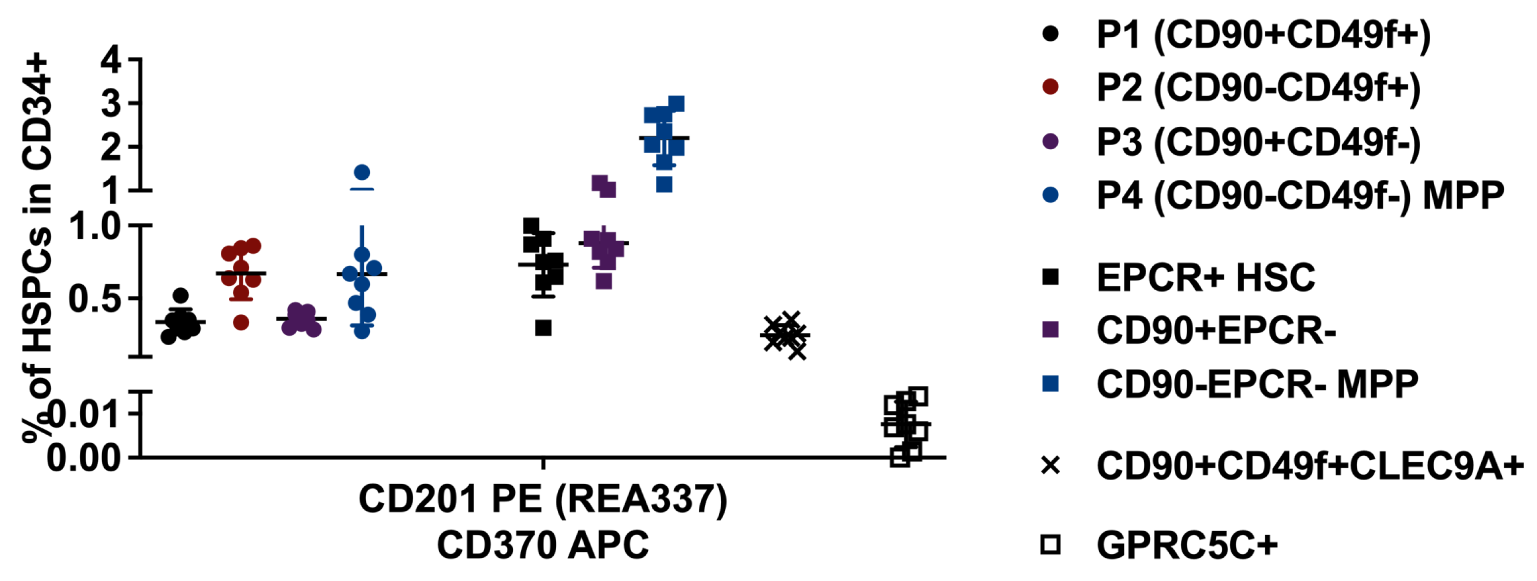


**Supplementary Figure E5.**

Percentage of the different CD34^+^CD38^-^CD45RA^-^ HSPCs cells in the CD34^+^ population using the depicted antibody clones and their respective conjugates (n=8 independent single CB). Of note, little to no CD90^+^CD49f^+^GPRC5C^+^ were detected as most of the CD34^+^GPRC5^+^ cells were CD38^+tohi^.
